# CAALYX: a new generation of location-based services in healthcare

**DOI:** 10.1186/1476-072X-6-9

**Published:** 2007-03-12

**Authors:** Maged N Kamel Boulos, Artur Rocha, Angelo Martins, Manuel Escriche Vicente, Armin Bolz, Robert Feld, Igor Tchoudovski, Martin Braecklein, John Nelson, Gearóid Ó Laighin, Claudio Sdogati, Francesca Cesaroni, Marco Antomarini, Angela Jobes, Mark Kinirons

**Affiliations:** 1 Faculty of Health and Social Work, University of Plymouth, Drake Circus, Plymouth, Devon PL4 8AA, UK; 2 Location Based Information Systems, Information and Communication Systems Unit, INESC Porto, Campus da FEUP, Rua Dr. Roberto Frias, 378, 4200-465 Porto, Portugal; 3 Telefónica Investigación y Desarrollo, Parque Tecnológico de Boecillo, 118-120, 47151 – Boecillo, Valladolid, Spain; 4 Corscience GmbH & Co. KG, Henkestr. 91, 91052 Erlangen, Germany; 5 Department of Electronic & Computer Engineering, College of Informatics & Electronics, University of Limerick, Limerick, Ireland; 6 Department of Electronic Engineering, National University of Ireland, Galway, Galway, Ireland; 7 COOSS Marche Onlus, Dipartimento R&D, via Saffi n° 4, 60121 Ancona, Italy; 8 Synkronix IIIncorporation Ltd, Dunston House, Dunston Corner, Hemingstone, Ipswich IP6 9QD, UK; 9 Department of Ageing & Health, St Thomas' Hospital (NHS), London SE1 7EH, UK

## Abstract

Recent advances in mobile positioning systems and telecommunications are providing the technology needed for the development of location-aware tele-care applications. This paper introduces CAALYX – Complete Ambient Assisted Living Experiment, an EU-funded project that aims at increasing older people's autonomy and self-confidence by developing a wearable light device capable of measuring specific vital signs of the elderly, detecting falls and location, and communicating automatically in real-time with his/her care provider in case of an emergency, wherever the older person happens to be, at home or outside.

## Background

Back in January 2003, we explored the concept of 'location-based health information services', and presented it as a new paradigm in personalised health information delivery [1]. Since then, location-aware applications and services, and location-sensitive mobile devices have undergone major improvements, and have become more widely available and less expensive [2]. These rapid developments have made their use in mission-critical tele-care and healthcare delivery significantly more prevalent today than it was in the near past [3]. A recent conference workshop, Locare '06, was fully dedicated to the subject of 'location-based services for healthcare' [4].

The year 2007 has been described as 'the year of Global Positioning System (GPS)-enabled mobile phones' par excellence [5,6]. In fact, mobile phones lend themselves very well to location-based technologies, as David Sym-Smith cleverly notes: "*Prior to mobile phones, the most common opener to a telephone conversation was 'How are you?' Today, on a wireless phone, more conversations open with 'Where are you?'*"[7] Such GPS-enabled mobile phones (GPS-enabled cameras and other gadgets also exist) will not just enable millions of people to collectively annotate the Earth in ways never done before [8], but will also open many other exciting and much needed location-based service possibilities and opportunities. On such an opportunity is perfectly exemplified in CAALYX [9].

## What is CAALYX?

CAALYX (Complete Ambient Assisted Living Experiment, 1st January 2007–31st December 2008) is a two-year project partially funded by the European Commission (EC) under the Sixth Framework Programme (FP6 – contract number IST-2005-045215 – in response to Strategic Objective: eInclusion Call 6). The total EC contribution to CAALYX is €1,850,000.00 Euros.

The project has a total of eight participants in six European countries: Telefónica Investigación y Desarrollo, Spain (Coordinator), Instituto de Engenharia de Sistemas e Computadores do Porto, Portugal, Corscience GmbH & Co KG, Germany, COOSS Marche Onlus, Italy, University of Plymouth, United Kingdom, Guy's and St Thomas' Hospital (NHS), United kingdom, Synkronix Ltd, United Kingdom, and University of Limerick, Ireland, with the National University of Ireland, Galway, Ireland, who for the purposes of this project, is an affiliate of the University of Limerick.

CAALYX aims at increasing older people's autonomy and self-confidence by developing a wearable light device capable of measuring specific vital signs of the elderly, detecting falls, and communicating automatically in real time with his/her care provider in case of an emergency, wherever the elderly person happens to be, at home or outside. Specifically, CAALYX's objectives are:

• To identify which vital signs and patterns are most important in determining probable critical states of an elder's health;

• To develop an electronic device able to measure vital signs and to detect falls of the older person in the domestic environment and outside. This gadget will have a geo-location system so that the monitoring system may be able to know the elder's position in case of emergency (especially outdoors);

• To allow for the secure monitoring of individuals organised into groups managed by a caretaker who will decide whether to communicate events identified by the system to the emergency service (112); and

• To create social tele-assistance services that can be easily operated by the users.

**Figure 1:**
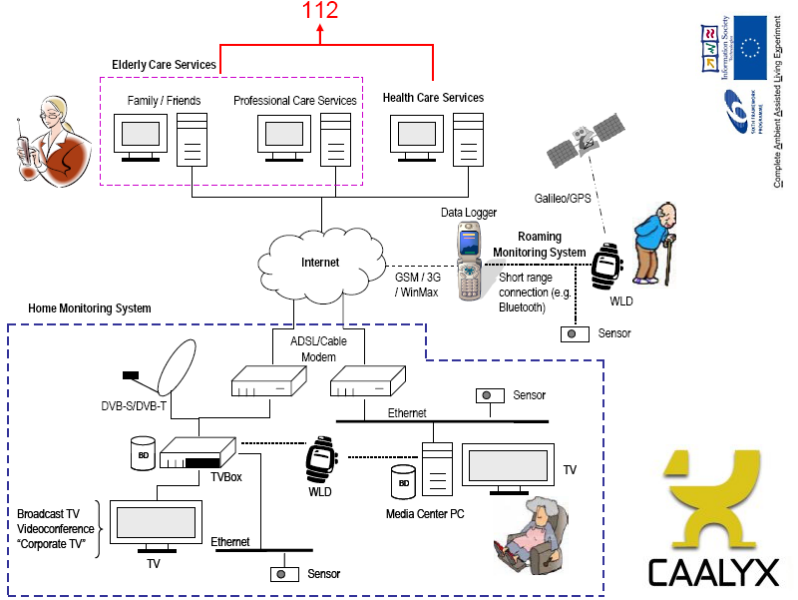
**CAALYX system diagram**. Diagram showing the main components of CAALYX (WLD = Wearable Light Device).

CAALYX considers three main areas of development: the 'Roaming Monitoring System', the 'Home Monitoring System' and the 'Central Care Service and Monitoring System' (Figure 1):

• **The Roaming Monitoring System **intends to monitor unobtrusively the older person when carrying out his/her daily activities in an independent way, both in the home and outdoors. Several vital signs besides falls will be measured and automatically communicated together with his/her geographic position to the Central Care Service in case of emergency, so that a rescue unit can be dispatched in a timely manner.

• **The Home Monitoring System **intends to extend the monitoring in the home environment, integrating other monitoring devices and sensors, as well as to integrate home automation devices in the system. Another important aspect is the support for video communication and VoIP (Voice over IP) using the TV set, which can also be used for remote monitoring and service-provision. This platform opens new possibilities for on-demand services for the elderly, like grocery shopping, cleaning, housekeeping or gardening, and periodic consultation with the doctor or personal caretaker. A key goal is to ensure social inclusion through the provided technology, e.g., video conferencing with friends and relatives, and ensuring local events awareness.

• **The Central Care Service and Monitoring System **will receive alerts from subscribed elder persons. The caretaker will evaluate whether received alerts need to be communicated to the emergency service (112), in which case the geographic position and data about the likely type of emergency (fall, stroke, etc.) will be provided to the emergency service, so that a suitably equipped emergency team may be dispatched in a timely manner to the patient's location. Besides this service, video-communication with the home environment will be provided to attend to the older person's demands. Other possible services include reminders to take pills, activity and scheduled visit reminders, e-visits, etc.

CAALYX uses a bottom-up design approach with full user representatives' involvement to satisfy final and intermediate user needs. Further details about CAALYX, including a typical Case Study illustrating the above functions in everyday use, are presented in the online public project presentation [10].

## Why CAALYX?

**Figure 2:**
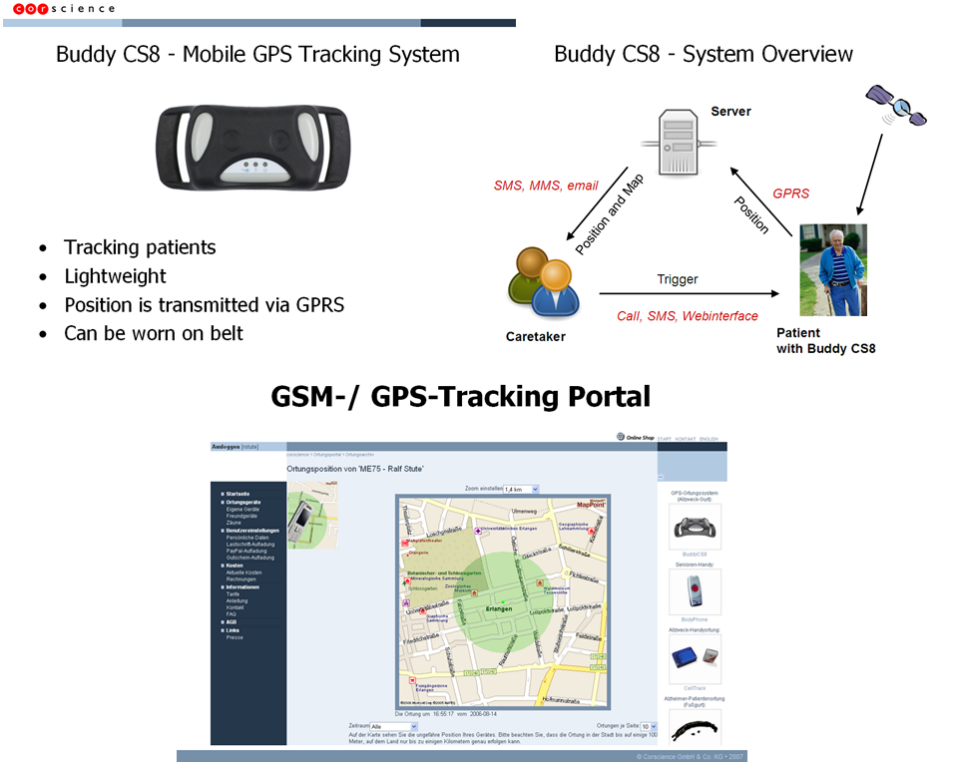
**Corscience mobile GPS tracking system**. Diagram showing the main components of Corscience mobile GPS tracking system.

Other tele-monitoring projects either send location information without much or any details about the current medical status of the user, e.g., the Columba Bracelet, a GPS-enabled bracelet permitting the localisation of Alzheimer patients if they get lost or become disoriented [11], and location-tailored health information services('push services') [12], or send detailed information about user's medical status without any knowledge about their location, e.g., M2DM – Multi Access Services for Telematic Management of Diabetes Mellitus [13], and MobiHealth [14]. However, to be able to offer to users proper help during emergencies wherever they might be (including incidents in which patients are unconscious or unable to adequately describe their location for any reason), a service would ideally require both detailed information about user's current medical status and details of the user's current location in order to dispatch a suitably equipped emergency team to the patient, and that is exactly what we are developing in CAALYX. This is helped by the fact that CAALYX does not rely on fixed fall sensors installed in one place (at home), like infrared fall detection sensors or cameras (computer vision) that are physically tied to, and configured for, an older person's residence and need to first learn about his/her daily routine movement, but uses novel wearable fall sensors (featuring integrated accelerometers and gyroscopes, developed at the University of Limerick, Ireland and at the National University of Ireland, Galway, Ireland, an affiliate of the University of Limerick for the purposes of this project). These wearable sensors accompany the older person anywhere they go, together with an outdoors' GPS solution. The latter will build on the industrial expertise and strengths of one of the project partners, Corscience GmbH & Co KG, Germany, in developing tracking systems (Figure 2) [15].

It is noteworthy that location sensitivity/awareness is now an FP7 (EU Framework Programme 7) trend/"requirement" for 'personalised health status monitoring' projects [16], so CAALYX and a few other projects like EmerLoc [3] are quite ahead of others in this respect.

## Current issues and limitations of the location-based/GPS features in CAALYX and some possible workarounds

### Failure to establish a GPS fix in difficult environments

The geo-positioning module in CAALYX might fail to establish a GPS fix when operating in some urban canyons/foliage/coated windshield environments, and indoors/inside buildings (e.g., inside a shopping mall). One possible workaround would be to develop (as part of a follow-on project) a more comprehensive and robust geo-positioning solution for mobile older people, combining multiple geo-positioning technologies to cover locations where conventional GPS alone would fail. Such a hybrid, "intelligent" solution should be able to *automatically and transparently *select and switch between multiple geo-positioning methods as appropriate/necessary to ensure uninterrupted location functionality. Other geo-positioning technologies to consider in this regard besides conventional GPS include:

• **Assisted GPS (A-GPS) and A-GPS over the Internet**: can speed up the time to first fix in difficult environments [17]; the area of coverage can also be extended by eliminating the need for downloading the ephemeris data directly from the satellite, which requires a higher signal quality than tracking alone;

• **WiFi Positioning System (WPS)**: SiRF and Skyhook have recently announced a hybrid GPS/Wi-Fi (WPS) location solution [18];

• **Rosum TV-GPS**: a TV based positioning solution with potential enhanced support for dense urban areas and inside buildings (currently only available for the North American/NTSC market) [19];

• **Cellular positioning**: offers a very wide coverage even in indoor situations while lacking the precision of satellite based navigation systems; and

• **Galileo Positioning System**: will not be fully rolled out until 2010. The Swiss company u-blox have recently announced the release of their first receiver for the European Galileo Positioning System, which will also use the signals of the American GPS satellites [20]. It is expected that Galileo will provide an enhanced coverage over the current GPS, higher positional accuracy, as well as better availability, making it possible to work in urban canyons and even in small buildings.

### Privacy issues

Location capability poses service providers with the challenge of responsibly handling consumers' personal privacy [1]. This is particularly important with 'tracking services' that continuously monitor and log user's location, like Wherifone, an American location-tracking service for the elderly and children [21], and other live tracking services using technologies like the GpsGate Server [22]. Such services raise many privacy concerns and questions; for example, "*If a consumer service allows one party access to the location of a second party, should that second party be notified when this location information has been provided?*"[23]

However, CAALYX's approach to location information privacy is different. CAALYX is an extensible user health monitoring platform that uses GPS as to support that function (health monitoring) and for emergency handling. Thus CAALYX is not continuously tracking older people, or continuously communicating their location in real-time with the central monitoring station. There are a number of reasons for this. Firstly, allowing the data logger (a mobile smartphone that users carry on them) to collect the data rather than continuously stream it to a remote server means that expensive bandwidth is saved. It is also far more power-efficient than a system that has to continuously transmit data and pick up real-time geographic information via GPS, a paramount feature in any handheld device. But most importantly, it means people will not feel as if their every move is being watched. Location information is only sent when required during an emergency or when an alarm is raised. As such CAALYX has the potential of setting the standards and providing a 'modus operandi' or 'best-practice' model for wireless location privacy in mobile, location-intelligent/enabled e-health services.

## Conclusions and future directions

Europe is about to face a significant social change, brought about by an unprecedented demographic change: the ratio of older people to the entire population is steadily growing, while the ratio of younger age groups, especially the working population (including healthcare workers) is shrinking. This demographic trend makes it difficult to foresee how Europe will find enough people to take care of its older population, without a major change in traditional older people's care methods. The role of e-health in facing such challenges will be very significant. In fact, leading health informatics think tanks like Professor Enrico Coiera are anticipating a total "reinvention" of healthcare by the year 2020, as the already significantly strained national health systems face the increasing needs to treat proportionately more people, with more illness, using relatively less tax money and fewer healthcare workers [24].

In this context, CAALYX represents a unique and much needed mobile, location-based (geo-aware) e-health service. Older people's autonomy (duration of independent living) and self-confidence can be greatly increased by wearing a light device that can measure vital signs, detect falls and location, and automatically raise an alert to their care centre in case of an emergency. Other tele-monitoring projects either send location information without much or any details about the current medical status of the user, or send detailed information about user's medical status without any information about their location. However, to be able to offer users proper assistance during emergencies wherever they might be (including incidents in which patients are unconscious or unable to adequately describe their location for any reason), a service would ideally require both detailed information about user's current medical status and details of user's current location, and that is exactly what CAALYX is offering.

To further improve service safety and reliability, the CAALYX design team will be looking at a 'technical contingency plan' to deal with problematic situations and locations when/where, for example, geo-positioning/getting a GPS fix using standard GPS is not possible (perhaps consideration will be given to using some form of 'Assisted GPS' in such cases) and/or when communication between the older person and a central CAALYX monitoring station is lost for some reason (e.g., bad network connection or technical fault in remote server). Future follow-on services should have enough embedded "intelligence" to be able to *automatically *deal with such situations and switch to a suitable alternative plan.

Other possible future directions include developing value-added geo-services for niche markets; for example, for Alzheimer's patients a geo-reminder service can be developed based on Ludimate's Geo-minder [25] to help with their short-term memory problems. The current version of CAALYX offers essentially the infrastructure for creating such services, and some non-mobile/non-location-based reminders, e.g., daily drug reminders at home, will be included in the pilot. But a location-based reminder to assist Alzheimer's patients could be something like "*When I pass supermarket, remind me to buy vegetables*". Then upon arriving at the marked location, the mobile phone can play an alarm and display a stored text note or a voice note previously associated to that location. This is far more superior to simple time-based reminders, as it also considers the location-based context.

## Authors' contributions

MNKB conceived and drafted the manuscript. AR, AM and MEV contributed portions of the text and Figure 1. AB, RF, IT and MB contributed portions of the text and Figure 2. All authors contributed unique insight and technical know-how in their respective areas of expertise, and revised and approved the final manuscript.
